# On the road to replication

**DOI:** 10.15252/emmm.201505965

**Published:** 2016-01-19

**Authors:** John FX Diffley

**Affiliations:** ^1^ The Francis Crick InstituteClare Hall LaboratorySouth MimmsHertfordshireUK

**Keywords:** Cancer

## Abstract

The 2016 Louis‐Jeantet Prize for Medicine winner John Diffley tells the story of his path to characterise and understand DNA replication.

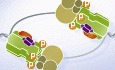

## An American in South Mimms

I had left New York a few days earlier—Halloween 1990—to start my new research group at the ICRF Clare Hall laboratories. It was Guy Fawkes Night at Clare Hall, which is located in the rural village of South Mimms just north of London. Guy Fawkes was a 17^th^ century religious zealot who tried unsuccessfully to blow up the Houses of Parliament; he was captured, tortured, and executed, events which are celebrated in Britain every November 5^th^ with fireworks and bonfires. I was standing in a soggy field, feet soaking wet and freezing in the drizzling rain, eating a cold sausage, and watching my new colleagues burn an effigy of our laboratory manager, Frank Fitzjohn, on the bonfire. I had clearly arrived in a foreign land!

At the time of my hiring, I was given the choice of the Clare Hall laboratories or the Lincoln's Inn Fields laboratories in central London. With LIF's reputation for cutting‐edge cancer research, and having lived in another big city, New York, for most of my life, friends and colleagues had expected me to choose LIF. Clare Hall was not yet the internationally recognised powerhouse of genome stability research it later became, but it was clear to me that I had found a home amongst a group of outstanding young biochemists including Rick Wood and Steve West. Soon Tim Hunt, Julian Blow, and Noel Lowndes joined the faculty, generating a vibrant atmosphere for cell cycle research. And, under the direction of Tomas Lindahl, the future of Clare Hall seemed very bright.

**Figure 1:**
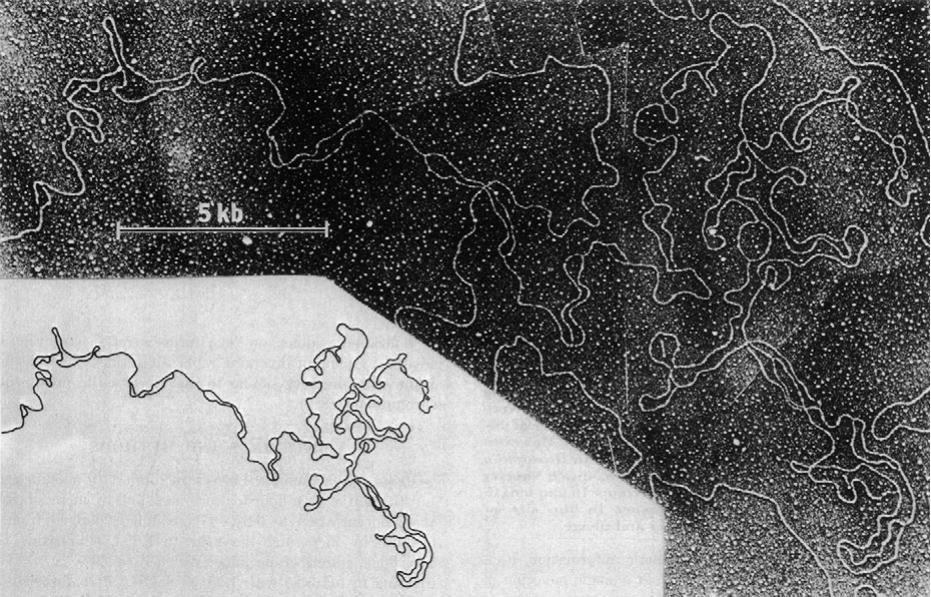
DNA replication bubbles from *Drosophila* cleavage nuclei This figure, reproduced with permission from Kriegstein and Hogness (1974), shows an electron micrograph (and accompanying trace) of DNA purified from very early (< 1 h) fertilised *Drosophila melanogaster* embryos. This particular molecule shows 23 replication bubbles in a region of 119 kb.

I had come to Clare Hall straight from a postdoc in Bruce Stillman's laboratory at Cold Spring Harbor. When I first arrived in Bruce's laboratory, he had just embarked on a major project to dissect cell extracts that supported the replication of SV40 DNA, and during my time at CSH, many of the “household names” in DNA replication such as PCNA, RPA, RFC were discovered. I, however, had a different agenda. As a student, I had been fascinated by electron micrographs of DNA from early *Drosophila* embryos (Fig 1) showing multiple replication “bubbles” along the chromosome (Kriegstein & Hogness, 1974). Although the idea that metazoan chromosomes were replicated from multiple replication origins had been demonstrated years earlier by fibre autoradiography, actually seeing these structures piqued my curiosity, and I became interested in the idea of trying to understand the events that led to the formation of these bubbles—the initiation of chromosomal DNA replication. For this, SV40 was not ideal since it relies on the viral‐encoded protein large T antigen (TAg) for origin recognition and replicative helicase activity. Little did I realise at the time that the “cellular TAg” I was chasing actually comprised some 32 gene products, and it would take us more than 25 years to reconstitute the initiation of DNA replication with purified proteins!

## The living years—characterising origins *in vivo*

**Figure 2:**
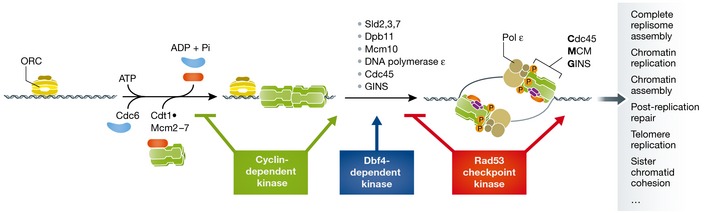
A model for DNA replication This model summarises some of our current understanding of how DNA replication initiates. In the first step, which is inhibited by CDK, ORC, Cdc6, and Cdt1 load the MCM helicase and an inactive double hexamer bound around double‐stranded DNA. In the second step, which is promoted by CDK, the listed firing factors, including the Dbf4‐dependent kinase, contribute to activating MCM by generating the Cdc4‐MCM‐GINS (CMG) holo‐helicase. This is followed by assembly of the complete replisome—the enzymes and other proteins that copy the genome. The DNA damage checkpoint kinase Rad53, when active, inhibits origin firing and stabilises stalled replication forks. Additional detail is found in the text.

My initial strategy was to use yeast replication origins, which had been identified years earlier, as a tool to identify origin‐binding proteins by biochemical approaches. Hopefully, we would be able to develop extracts that could replicate plasmids containing yeast origins, analogous to the SV40 system. Indeed, I spent many months making and testing extracts from S phase‐arrested cells for origin‐dependent incorporation of radio‐labelled nucleotides into DNA to no avail. Fortunately, by the time my postdoc was drawing to a close, I had been productive enough to convince ICRF to hire me, but it was clear to me that different approaches would be needed to crack this problem. In my new laboratory, I decided to establish techniques to look at proteins binding to replication origins *in vivo*. This was before chromatin immunoprecipitation reached the masses, and so we settled on developing genomic footprinting, a technique that uses DNase1 to probe for protection of DNA sequences on chromatin *in situ*. With this, my first student Julie Cocker and I soon had evidence that the essential “A element” in yeast origins was bound by a protein and was flanked by a distinctive set of DNase1 hypersensitive sites at 10‐bp intervals (Diffley & Cocker, 1992). In a biochemical tour de force (Bell & Stillman, 1992), Steve Bell in the Stillman laboratory had identified and purified a six subunit protein that specifically recognised the A element and generated a pattern of protection and hypersensitivity nearly identical to our *in vivo* pattern, and so ORC (Origin Recognition Complex) was born! Because our footprints were initially performed on asynchronous cells and the A element was completely protected, we suggested that ORC was likely to be bound at origins all or most of the cell cycle, and thus, its binding by itself was not likely to be the trigger for DNA replication. In 1994, using synchronous cell populations, we showed that ORC was indeed bound throughout the cell cycle, but during G1 phase, the ORC footprint was extended by an additional ~70 bp of protection, a complex we called the “prereplicative complex” or pre‐RC (Diffley *et al*, 1994). Over the next few years, we showed that the pre‐RC footprint required not only ORC, but also Cdc6, Cdt1, and the MCM complex (Fig 2). Pre‐RCs first assemble at origins right at the end of mitosis, coincident with the inactivation of cyclin‐dependent kinase (CDK), and in a collaboration with the laboratory of Kim Nasmyth (Dahmann *et al*, 1995), we showed that premature inactivation of CDK before anaphase was sufficient to promote pre‐RC assembly. This led us to propose the idea that CDK has a dual role in replication: On the one hand, it is required to promote DNA replication in S phase—as we would later show, by phosphorylating two key firing factors, Sld2 and Sld3 (Zegerman & Diffley, 2007). But on the other hand, it prevents pre‐RC assembly outside of G1 phase. Thus, pre‐RCs can only assemble during G1 phase when CDK activity is low; activation of CDK in S phase then both triggers DNA replication, and prevents re‐assembly of pre‐RCs at origins that have already fired. This idea neatly explained how multiple replication origins could be regulated to fire just once in each cell cycle (Fig 2).

It also helped explain why my early attempts to get S phase extracts to support replication failed: The hydroxyurea‐arrested budding yeast cells I had been using to make extracts have high CDK levels, which would block pre‐RC assembly. In addition to CDK, these cells also have high levels of the Rad53 DNA damage checkpoint kinase, which blocks origin activation (Santocanale & Diffley, 1998). So, the years we had spent trying to understand how events at replication origins are regulated *in vivo* were pointing us in the right direction, and by the close of the 1990s, we felt we knew enough to get back to the biochemistry.

## Can we build it? Yes we can!

The *in vivo* experiments had taught us that the initiation reaction would need to be reconstituted in two sequential biochemical steps: First, pre‐RCs would need to be assembled in the absence of CDK activity, and then, origin firing would require high CDK activity. We knew cells arrested in G1 phase were competent for pre‐RC assembly *in vivo*, but we also knew from Lucy Drury's work that the critical pre‐RC assembly factor, Cdc6, was highly unstable in G1 phase. So, Takashi Seki used extracts from G1‐arrested cells that conditionally overexpressed Cdc6 and showed that he could assemble MCM onto DNA in a Cdc6‐ and cell cycle‐dependent manner (Seki & Diffley, 2000). When Dirk Remus joined the laboratory, he performed mass spectrometry on pre‐RCs assembled in extracts and identified ORC, Cdc6, Cdt1, and MCM, but no additional proteins, suggesting we had the complete list of pre‐RC components. Dirk then purified these proteins and reconstituted the reaction. In collaboration with electron microscopists Fabienne Beuron and Ed Morris, Dirk showed that MCM is loaded as a head‐to‐head double hexamer and that this double hexamer is bound as topologically closed rings around double‐stranded DNA (Remus *et al*, 2009).

The next step was never going to be easy: Activation of the MCM helicase involves melting the DNA duplex, reopening the MCM ring, separation of the double hexamer into two single hexamers, extrusion of the lagging strand template from the interior and re‐closing of the ring around the leading strand template. We also knew this step requires a long list of firing factors. But we were developing effective workflows for expression and purification of replication factors, and Joe Yeeles, an experienced and talented biochemist, was soon in position to look for DNA replication. Then, one August afternoon in 2014, there it was: A small smudge of radio‐labelled DNA whose synthesis required everything we knew it should! After some optimisation, the replication products grew in length and partitioned into leading and lagging strand products (Yeeles *et al*, 2015)—it was clear we had a minimal DNA replication system up and running!

## How far can we take this?

We continue on our quest to reconstitute the entire DNA replication reaction with purified proteins, and we are already learning a great deal about initiation mechanism. In addition to this, though, I believe we can extend our biochemical approaches to understand how DNA replication interfaces with many nuclear processes, including epigenetic inheritance, chromosome cohesion, and post‐replication repair. Ultimately, we hope to contribute to the reconstitution of a functional chromosome from constituent parts. The next few years will be fascinating.

Finally, this was not meant as a review but rather as a personal account of the journey leading to our reconstitution of the replication initiation reaction, so I apologise to all those not mentioned in the ten allowed citations. I have been privileged to work with an amazing group of young scientists over the past quarter century, and I am grateful to them for sharing their talents and their enthusiasm with me. And, despite my initial reservations, I now cannot imagine November without Guy Fawkes Night!
